# Much More Than a Pleasant Scent: A Review on Essential Oils Supporting the Immune System

**DOI:** 10.3390/molecules24244530

**Published:** 2019-12-11

**Authors:** Agnes Peterfalvi, Eva Miko, Tamas Nagy, Barbara Reger, Diana Simon, Attila Miseta, Boldizsár Czéh, Laszlo Szereday

**Affiliations:** 1 Department of Laboratory Medicine, Medical School, University of Pecs, Ifjusag utja 13., 7624 Pecs, Hungary; tamasnagy01@gmail.com (T.N.); barbara.reger@freemail.hu (B.R.); miseta.attila@pte.hu (A.M.); czeh.boldizsar@pte.hu (B.C.); 2 Neurobiology of Stress Research Group, Szentagothai Research Centre, University of Pecs, Ifjusag utja 20., 7624 Pecs, Hungary; 3 Department of Medical Microbiology and Immunology, Medical School, University of Pecs, Szigeti ut 12., 7624 Pecs, Hungary; miko.eva@pte.hu (E.M.); szereday.laszlo@pte.hu (L.S.); 4 Department of Immunology and Biotechnology, Medical School, University of Pecs, Szigeti ut 12., 7624 Pecs, Hungary; simon.diana@pte.hu

**Keywords:** essential oils, herbal medicine, immune system, immune functions, immune enhancement, dietary supplementation, forest bathing, eucalyptus, ginger

## Abstract

The augmenting acceptance and application of herbal medicine in prevention and treatment of diseases also involve the use of plant essential oils (EOs) through different routes of administration (aromatherapy). Scientific data supporting the efficacy of certain herbal products are continuously growing; however, the cumulative evidence is not always sufficient. The anti-inflammatory properties of EOs have been investigated more extensively and also reviewed in different settings, but so far, our review is the first to summarize the immune-supporting properties of EOs. Our aim here is to synthesize the currently available data on the immune function enhancing effects of EOs. An online search was conducted in the PubMed database, which was terminated at the end of July 2019. Other articles were found in the reference lists of the preselected papers. Studies that applied whole EOs with known components, or single EO constituents under in vitro or in vivo laboratory conditions, or in human studies, and de facto measured parameters related to immune function as outcome measures were included. Two specific fields, EO dietary supplementation for livestock and fish, and forest bathing are also explored. Some EOs, particularly eucalyptus and ginger, seem to have immune function enhancing properties in multiple studies.

## 1. Introduction

**Figure 1:**
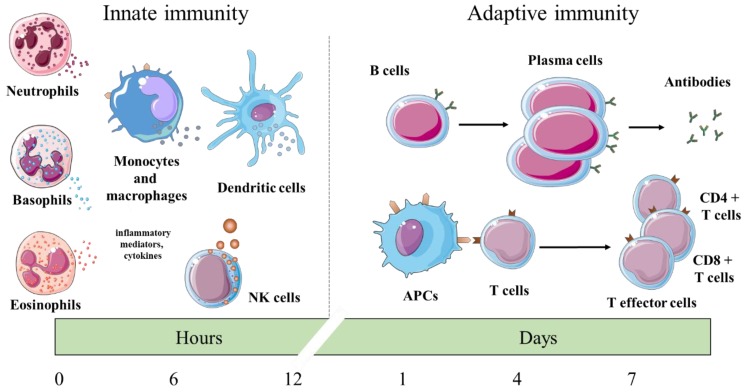
Timeline, principal cells, and mechanisms of the innate and adaptive immune responses. NK cells, natural killer cells; APCs, antigen-presenting cells; CD, cluster of differentiation.

Herbal medicine is receiving increasing attention and has been applied more extensively in disease treatments or promoting health during the last decades. A growing amount of data demonstrates the efficacy of herbal products, but in many cases the available evidences are still scarce. Certain plants possess immunomodulatory properties exerting effects on various parts of the immune system on both cellular and molecular levels: T cells and other immune effector cells, cytokine, and antibody production [1]. Immune responses can be classified into two categories: The innate (natural) and the adaptive (acquired) immune response. The innate immune response involves neutrophil, eosinophil and basophil granulocytes, mast cells, dendritic cells, monocytes and macrophages, and natural killer (NK) cells. Their functions include phagocytosis, release of inflammatory mediators, cytokine production, and antigen presentation. The adaptive response involves the immunoglobulin (Ig)/antibody-producing and secreting B cells as plasma cells, and T cells including CD4+ T helper cells and CD8+ cytotoxic T cells. Innate immunity provides the first reaction of the immune response, while adaptive immune responses require more time for the activation of various lymphocyte subpopulations. Figure 1 shows the timeline during innate and adaptive immune responses (Figure 1). Cytokines are a diverse group of low-molecular-weight soluble proteins regulating cellular proliferation, differentiation, and activity [2,3]. CD8+ cytotoxic T cells and NK cells destroy infected and malignant cells by inducing their apoptosis. One pathway is by secreting the pore-forming perforin, granzyme proteases, and granulysin from cytolytic granules toward the target cells [4,5]. Stimulation of the immune system relates to the non-specific activation of the innate and/or adaptive immune responses leading to enhancement of certain immune functions, which are protecting against various pathogens and malignantly transformed cells [6,7].

It is known that whole plants, herbs, their parts or extracts, or other herbal products may all have immunostimulatory properties, like *Echinacea* (purple coneflower), licorice root (*Glycyrrhiza glabra*), garlic, and two species of cat’s claw, *Uncaria guianesis* and *Uncaria tomentosa*. The mechanism of immune enhancement was either promoting the activity of lymphocytes, increasing phagocytosis by macrophages, inducing interferon production, or stimulating NK cell activity [8]. Among the immunomodulatory activities of *Echinacea purpurea*, activation of monocytes/macrophages, polymorphonuclear leukocytes, and NK cells has been found [9,10]. Hydrolyzate proteins of Changbai Mountain walnut (*Juglans mandshurica*) administered orally to mice could boost the immune system by improving spleen lymphocyte proliferation and macrophage activity, increasing the number of CD4+ and CD8+ T cells, IgA and secretory IgA levels, and interleukin (IL)-6 and interferon gamma (IFNγ) expression and mRNA levels [11].

Essential oils (EOs) are highly concentrated, aromatic volatile oils of plant origin with numerous chemical constituents that are extracted by steam distillation, hydrodiffusion, or pressure [12]. Aromatherapy is a field of complementary medicine that uses EOs to treat and prevent diseases via several routes of administration: Usually topical, massage, inhalation, or oral [13]. Antimicrobial, anti-inflammatory, antioxidant, anticancer, anxiolytic, antidepressant, analgesic, and antidiabetic activities of EOs have been studied and reported recently [14,15,16,17,18,19,20]. Scientific evidence on the immunostimulatory effects of EOs is rather fragmentary, but there seems to be a potential for certain EOs to be able to enhance some immune functions. Standen and Myers conducted a survey among Australian aromatherapy educators asking them to recommend EOs that are regarded as immune-stimulating (or, in a separate question, that are anti-inflammatory). The authors found limited consensus amongst them, and between these and both the popular authors and the few published scientific studies [21]. More research is justified to be done in this area to increase the body of evidence, and an overview of currently available data is relevant.

The objective of this paper is to review currently available data on EOs augmenting beneficial, measurable immune function parameters by summarizing major developments in the wider field. In our literature search, we applied the following keywords and used the PubMed database: “essential oil immunostimulatory”, “essential oil immunomodulatory”, and “essential oil immune”. Other articles were found in the reference lists of the preselected papers. The search was terminated at the end of July 2019. Any work was considered for inclusion that applied whole EOs with known components, or single EO constituents from any common, or less well-known plants under in vitro or in vivo experimental conditions, or in human studies, and de facto measured some immunological parameters related to immune function as outcome measures, like phagocytic activity, lymphocyte proliferation, secretion of cytokines, production of immunoglobulins, hemagglutination titers, delayed-type hypersensitivity, expression of activation markers, or NK cell cytotoxicity. The focus of this review is immune function enhancement, though sometimes negative, opposite or inconsistent results are also mentioned in order to offer a more nuanced view. Two further specific fields of immune function enhancement with EOs are explored. One is the immunomodulatory (and other) effects of the dietary supplementation of livestock and fish with EOs or EO components. The other is forest bathing, whose health-promoting and immune function enhancing effect may be mediated by wood EOs present in the forest air. Table 1 summarizes the EOs used in the respective publications.

## 2. In Vitro Investigations

Research on the effects of EOs on immune cell lines, or cells derived from animals (mice, rats) or humans untreated with EOs are considered as in vitro investigations. Eucalyptus (*Eucalyptus globulus*) EO stimulated the phagocytic activity of cultured human monocyte-derived macrophages (MDMs) after 24 h treatment, independently of concomitant lipopolysaccharide (LPS) treatment [23]. The authors also tested lavender and tea tree EO to exclude a non-specific stimulatory effect caused by the oil preparation, but none of the oils affected MDMs phagocytic activity. In the same experiment, pro- and anti-inflammatory cytokines produced by the activated macrophages were also assessed. Their levels were not significantly different from the untreated control, and were significantly lower (IL-4, IL-6, tumor necrosis factor alpha (TNFα)) if a 24 h EO treatment prior to the addition of LPS to cell culture was compared to LPS treatment alone [23]. The authors suggest that the different mechanisms, and possibly different phagocytic receptors (complement versus Fc receptors) involved in the EO or LPS induced internalization may explain the increased phagocytosis coupled to a low release of mainly pro-inflammatory cytokines with the eucalyptus EO, as EO-stimulated phagocytosis required integrity of the microtubular network, while LPS stimulation did not [23]. On the other hand, anti-inflammatory properties of eucalyptus EO or one of its major components, 1,8-cineole have also been reported. The inhibition of TNFα and IL-1β production and decreased nuclear factor kappa B (NFκB) activity were also observed in human monocytes [24].

EO from the leaves of *Schinus molle* increased TNFα and nitric oxide production, reduced IL-10 production, and generated no change in the levels of IL-6 compared to negative controls in cultures of human lymphocytes and activated macrophages, altogether leading to an activation of the immune system [31]. EO extracts from various parts of *Ferula iliensis* dose-dependently enhanced superoxide anion production in isolated human neutrophils and bone marrow leukocytes from mice. The EOs stimulated calcium influx by activation of TRPV1 channels since capsazepine, a TRPV1 channel antagonist was found to inhibit its activation [27]. Garlic (*Allium sativum*) EO and some of its pure organosulfur compounds augmented functional responses in isolated human neutrophils, resulting in increased calcium flux and/or reactive oxygen species (ROS) production [22].

Frankincense EO (derived from *Boswellia carterii*) showed strong immunostimulant effect when based on lymphocyte proliferation assay. Human peripheral venous blood lymphocytes were stimulated with the mitogen phytohaemagglutinin (PHA), and the mitogenic response induced in the presence of frankincense EO (90% lymphocyte proliferation) was comparable to that of known immunostimulants like *Echinacea purpurea* aqueous extract (95%) and levamisole (85%) [28]. Thyme (red) EO from *Thymus vulgaris* significantly enhanced, in vitro, the intracellular killing of *Candida albicans* by separated human polymorphonuclear granulocytes in comparison with EO-free controls and was comparable to fluconazole used as a positive control [33]. *Pituranthos tortuosus* EO at certain concentrations was able to induce the proliferation of splenocytes from naïve mice and also significantly enhanced LPS-stimulated splenocyte proliferation, which is according to the authors “suggestive of a potential for activation of B cells and enhanced humoral immune responses” [30].

Rodrigues and colleagues [32] searched for possible mechanisms that may mediate the anti-Leishmania effects of *Syzygium cumini*. *S. cumini* leaf EO and its major component α-pinene increased the phagocytic (phagocytosis of neutral red-stained zymosan) and lysosomal (uptake of the cationic dye neutral red) activities and also the nitric oxide production of non-stimulated mouse peritoneal macrophages [32].

*Lavandula angustifolia* EO increased the phagocytic rate and stimulated the containment of intracellular bacterial replication in cultured human MDMs pretreated with the EO and then infected with *Staphylococcus aureus*. This stimulation was coupled with the expression of genes involved in ROS production. Parallelly, the EO treatment repressed major pro-inflammatory cytokines (IL-1, IL-6) exerting an anti-inflammatory effect [29]. That is, similarly to the effects of eucalyptus EO in the work by Serafino et al. [23], lavender EO in the study of Giovannini et al. [29] enhanced the innate immune response by stimulating phagocytosis but also mitigated a consequent inflammatory response thus supporting and balancing the overall immune reaction.

Some inconsistent results are also available in the literature. Eugenol, a component of clove (*Syzygium aromaticum*) EO, after in vitro treatment of mouse peritoneal macrophages, suppressed nitric oxide release in LPS-stimulated macrophages, but low doses enhanced nitric oxide production by unstimulated cells. TNFα release was suppressed at all doses, while IL-6 release was stimulated in LPS-treated cells. Effects on IL-12 production seemed to depend on the concentrations and on the activation state of the macrophages [25]. In another experiment, eugenol, when used to treat mouse splenocytes in vitro, reduced PHA-stimulated splenocyte proliferation, but enhanced LPS-stimulated cell expansion. IFNγ release was suppressed, and IL-4, IL-10, and transforming growth factor beta (TGFβ) secretions were induced in splenocytes suggesting, according to the authors, that eugenol could enhance the humoral immune response by shifting the cytokine pattern toward T helper 2 responses [26].

Research on the immunomodulatory effects of EOs and pure compounds regarding innate immune responses may be enhanced by the method recently developed by Lang and colleagues, who adapted high content analysis for the monitoring of immunomodulatory activities of EOs enabling the simultaneous evaluation of phagocytosis, production of inducible nitric oxide synthase (iNOS) and secretion of IL-6 in the same experimental design, on the same batch of cells [66].

## 3. In Vivo Pre-Clinical Studies

The immunomodulatory effects of clove (*Syzygium aromaticum*), ginger (*Zingiber officinale*), and sage (*Salvia officinalis*) EOs were assessed by Carrasco et al. in healthy and cyclophosphamide-immunosuppressed mice administered the EOs orally, by determining the antibody titers with hemagglutination (humoral immune response) after immunization with sheep red blood cells (SRBCs), and measuring paw volume after challenging with a second SRBC suspension (delayed-type hypersensitivity, cellular immune response) [41]. Clove EO increased the leukocyte number, augmented the delayed-type hypersensitivity (DTH) response, while in immunosuppressed mice, it restored cellular and humoral immune responses. Ginger EO was also able to improve the humoral immune response in immunosuppressed mice. Sage EO demonstrated no immunomodulatory activity [41]. Components of *Carum copticum* seeds EO administered orally to mice were tested by measuring primary (on day 14) and secondary (on day 21) antibody levels following immunization with SRBCs, by assessing foot-pad reaction after an injection of SRBCs in the paw on day 22 (DTH), and by examining phagocytosis of heat-killed *Candida albicans* cells by peritoneal macrophages on day 25. The administration of α-pinene and carvacrol augmented the primary and secondary antibody syntheses, DTH-response, and phagocytosis, thus displaying an overall stimulatory effect [34].

The effects on phagocytic activity of eucalyptus (*Eucalyptus globulus*) EO were investigated by Serafino et al. [23]. EO in drinking water was orally administered to immuno-competent rats in the absence or the presence of immunosuppression induced by 5-fluorouracil (5-FU), and then peripheral blood monocytes/granulocytes were isolated, as well as MDMs were cultured ex vivo to perform phagocytosis tests. A 15-day long EO treatment significantly increased the percentage of circulating monocytes and their phagocytic activity in EO treated rats compared to EO untreated controls without effect on granulocytes or lymphocytes. This stimulatory effect on phagocytic activity was also retained in MDMs. EO treatment also inhibited the 5-FU induced myelotoxicity and restored the granulocytes/monocytes’ and MDMs’ phagocytic ability [23,24].

Halder et al. [35] explored the effects of the EO isolated from the buds of *Eugenia caryophyllata* on humoral immunity (by measuring the hemagglutination titers to SRBCs) and on cell-mediated immunity (by assessing foot-pad thickness after sensitization and later challenge by keyhole limpet hemocyanin) in rats following daily intraperitoneal EO administration. Both the primary (on day seven) and secondary (on day nine following a booster dose of antigen on day seven) antibody levels increased compared to untreated controls augmenting humoral immunity, unlike cell-mediated immunity, which decreased as shown by a reduction in foot-pad thickness when tested after 14 days of EO administration [35]. Orange EO significantly increased the level of IgA in serum, limonene significantly increased the levels of IgA, IgG, and IL-2 in serum, and linalool raised IgA and IgG after 28 days of intragastric administration for mice in a work by Wang and colleagues. Limonene and linalool (and also citral) were examined as components of orange EO [39].

Massoia (*Massoia aromatica*) bark infusion containing in low doses C-10 massoialactone, the major component of massoia bark EO, was administered orally to rats every day for 14 days in a study conducted by Hertiani et al., and the effects on the phagocytic activity of peritoneal macrophages were evaluated [36]. In a previous in vitro experiment massoia bark EO and C-10 massoialactone were found to enhance the phagocytic activity of mouse macrophages, and the phagocytosis stimulation was correlated to the massoialactone content [37]. Following the treatment with massoia bark infusion, the phagocytic activity of macrophages significantly increased in rats [36].

Immune responses of mice injected intraperitoneally with EO of niaouli (*Melaleuca viridiflora*) once a day for nine days from the next day of immunization with keyhole limpet hemocyanin (KLH) were investigated by Nam and colleagues [38]. They revealed overexpression of CD25 (an activation marker) on freshly isolated draining lymph node T cells, but not on B cells. KLH stimulated proliferative response and IFNγ production of T cells ex vivo in the niaouli EO treated group. The EO did not affect KLH-specific immunoglobulin levels in mice. Finally, higher production of TNFα and IL-12 was demonstrated by splenic macrophages from EO-treated mice when stimulated with LPS and IFNγ. The authors conclude that niaouli EO “potentiates T cell-mediated cellular immunity and macrophage activity” [38].

Reyes et al. examined the protective effects and possible mechanisms of action of the EO from Korean red ginseng (*Panax ginseng*) against *Brucella* infection in mice [40]. Ginseng extracts were previously shown to enhance phagocytic activity of macrophages in several studies [67]. Red ginseng EO after 14 days of oral treatment led to increases in the serum levels of TNFα and IFNγ in the uninfected but red ginseng EO-treated group of animals compared to the negative controls thus stimulating an immune response as suggested by the authors [40].

*Zanthoxylum rhoifolium* leaf EO and one of its major constituents, β-caryophyllene, significantly increased the survival of Ehrlich ascites tumor-bearing mice treated intraperitoneally for four days from the next day after tumor inoculation in a study by da Silva and co-workers [42]. When investigating the possible mechanism of the anti-tumor effect, the EO and β-caryophyllene did not show direct anti-tumor activity in vitro, but they significantly enhanced NK cell cytotoxicity against Ehrlich ascites cells and also against mouse T cell lymphoma cells (YAC-1) in treated compared to untreated mice [42].

Some papers demonstrate no immunomodulatory effects of certain EO components. In a principally toxicological study, when investigating the effects of three different doses of safranal, a major constituent of *Crocus sativus* EO, in mice, no significant changes or any marked effects could be induced in immune system parameters of mice regarding spleen cellularity, hemagglutination titers after immunization with SRBCs, DTH response, cytokine (IFNγ, IL-4) production of splenocytes, and lymphocyte proliferation to PHA [68].

## 4. Dietary Supplementation for Livestock and Fish

The use of antibiotic growth promoters for the livestock has been banned in the European Union since 2006 [69]. The use of EOs instead as feed additives has gained attention in recent years as a possible natural alternative. In general, EOs seem to benefit the digestive system and improve nutrient absorption, reduce the number of pathogens in the gut, have antioxidative capacity, and improve the immune system [69]. EO supplementation has been reported to improve the immune status of weaned pigs, e.g., increased lymphocyte proliferation rate, phagocytosis rate, and higher serum levels of IgG, IgA, IgM, complement (C)3, and C4 were observed [69]. Several studies examining the impact on weaned piglets apply a ready-to-use EO product, a blend containing a certain amount of thymol and cinnamaldehyde [45,46,47,48].

In broiler chickens, the dietary supplementation of a blend of thyme (*Thymus vulgaris*), peppermint (*Mentha piperita*), and eucalyptus (*Eucalyptus globulus*) EOs at 40:40:20 ratios at increasing dosages linearly enhanced the antibody titers in serum to infectious Newcastle, bronchitis, and bursal disease viruses on day four after vaccination. On day eight after inoculation, the antibody titers at certain EO blend dosages were significantly higher compared to the control group [43]. Though often a mixture of EOs or of various compounds are applied under non-laboratory conditions, the results of the experiments assessing the effects of single EOs or components may yield valuable information or offer guidance for the conduction of in vitro, in vivo, and finally, human studies, to eventually promote human health.

Eucalyptus (*Eucalyptus globulus*) EO supplementation evoked the greater primary antibody response (IgM) to SRBCs in broiler chickens compared to those on control diet, but the secondary antibody response (IgG) did not differ significantly [51]. Thyme (*Thymus vulgaris*) EO supplementation increased IgA concentration in duodenal mucosa, and stimulated the phagocytic activity of blood polymorphonuclear cells of broiler chickens [56]. Savoury (*Satureja hortensis*) EO dietary supplementation in certain concentrations significantly increased serum total immunoglobulin and serum lysozyme activities in angelfish (*Pterophyllum scalare*) compared to the control group [55]. Dietary supplementation of equal levels of thymol and carvacrol to juvenile hybrid tilapia fish (female *Oreochromis niloticus* × male *Oreochromis aureus*) enhanced the phagocytic activity of head kidney macrophages and the plasma lysozyme activity [44]. American basil (*Ocimum americanum*) as a feed additive increased hemolytic activity of the complement system of red drum (*Sciaenops ocellatus*) [52]. *Ducrosia anethifolia* EO dietary supplementation with different levels and durations led to higher white blood cell counts, macrophage activity (respiratory burst activity), and IL-1β and TNFα gene expression levels in kidney and spleen in rainbow trout (*Oncorhynchus mykiss*) [50].

Besides a number of studies indicating the immunostimulatory effects of different EOs or components in various animal species, there are also investigations showing no effects of EOs on the examined immune functions. Dietary supplementation of oregano (*Origanum vulgare*) EO to sows during lactation led to greater number of T lymphocytes in the milk but had no effect on immune responses (immunoglobulin concentrations, T lymphocytes, and NK cell activity) in suckling piglets [54]. Another oregano (*Origanum onites*) EO supplementation to broiler chickens neither exerted significant effects on immune responses (serum IgG, IgM titers, and antibody titer against the Newcastle Disease Virus) [53]. Garlic (*Allium sativum*) or juniper berry (*Juniperus communis*) EOs supplemented to midlactating Holstein cows did not affect the total and the differential numbers of white blood cells, or the concentrations of serum amyloid A and haptoglobin in the plasma [49]. An additional nuance raised by the nutrigenomics study of Sabino et al. is the sex-dependent effect on the liver and muscle expression profile of genes mainly involved in immune, inflammatory, and stress pathways in male and female lambs fed with a diet supplemented with a mix of cinnamon bark, dill seed, and eucalyptus leaves EOs [70].

## 5. Human Studies

Studies examining the effects of EOs on the immune functions of healthy human subjects are scarce, not to mention the investigation of effects in different medical conditions.

Komori et al. [61] explored the effects of citrus fragrance on immune function in conjunction with depressive disorder. Citrus fragrance, which was created by mixing lemon oil with several other materials such as orange oil, bergamot oil, and *cis*-4-hexenol, was applied to 12 patients with major depressive disorder until remission (four to 11 weeks) through olfactory stimulation; the dosages of antidepressants could be reduced significantly. The citrus fragrance also affected CD4/CD8 T cell values and NK cell activity, which returned to almost the normal range suggesting a restoring effect of the fragrance on immune function [61].

After the exposure of 12 healthy male subjects to vaporized hinoki cypress (*Chamaecyparis obtusa*) stem EO for three consecutive nights in an urban hotel room, NK activity, the percentages of NK cells, and perforin, granulysin, and granzyme A/B-expressing cells were found to be significantly increased in peripheral blood, while the percentage of T cells significantly decreased in a work by Li et al. [60].

In a study conducted by Chen et al. [64], 24 healthy pregnant women received 70 min of whole body aromatherapy massage with 2% lavender (*Lavandula angustifolia*) EO blended with almond oil every other week between 16 and 36 weeks gestational age, 10 times in total. Salivary IgA was used as an indicator of immune function, and levels were measured before and after each aromatherapy session. The pregnant women in the massage group had significantly higher IgA levels immediately after the aromatherapy massage compared to the control group (28 pregnant women who received only routine prenatal care). Regarding the longitudinal effects, the salivary IgA levels before the massage at 32 and 36 gestational weeks were significantly higher than the IgA before the massage at 16 weeks (baseline). According to the authors’ interpretation, the aromatherapy massage could significantly enhance immune function in pregnant women [64]. Takeda and colleagues [58] found that salivary IgA concentrations increased significantly as compared to the initial values after 45 min of aromatherapy massage with EOs diluted in carrier oil, as well as after a massage treatment with carrier oil alone, or also after rest. For the aromatherapy massage orange sweet (*Citrus sinensis*), true lavender, and marjoram sweet (*Origanum majorana*) EOs were blended and macadamia nut (*Macadamia integrifolia*) oil was used as the carrier oil; 13 healthy volunteers participated in the study [58]. On the other hand, no significant difference in salivary IgA levels between aromatherapy massage and massage with carrier oil alone was reported by Kuriyama et al. [57]. Eleven healthy volunteers were recruited in the study; massage sessions lasted for approximately 30 min, and a combination of lavender (*Lavandula angustifolia*), cypress (*Cupressus sempervirens*), and sweet marjoram (*Origanum majorana*) EO in sweet almond (*Prunus dulcis*) oil was used for aromatherapy massage [57]. In the same work, aromatherapy massage (but not the control massage) was demonstrated to induce a significant increase in the numbers of peripheral blood lymphocytes, CD8+ T cells, and CD16+ (NK) cells [57].

Sixty-six early stage colorectal cancer patients receiving adjuvant chemotherapy were enrolled in a randomized controlled trial conducted by Khiewkhern and co-workers [62]. The aromatherapy massage for the intervention group consisted of three, 45 min light Thai massage sessions over one week with ginger EO in coconut oil, while the control group received standard supportive care only. At the end of the week, in the massage group the mean lymphocyte count was significantly higher, the symptom severity scores for fatigue, pain, and stress were significantly lower than in the control group [62]. Statistically significant increases in the numbers of peripheral blood leukocytes and lymphocytes were reported by Imanishi et al. in a trial that included 12 breast cancer patients, who received a 30 min aromatherapy massage twice a week for four weeks using sweet orange (*Citrus aurantium*), lavender (*Lavandula angustifolia*), and sandalwood (*Santalum album*) EOs in jojoba oil [59].

Trellakis and colleagues examined the potential immune effects of short-term olfactory exposure to different EOs in 32 blinded healthy subjects (16 male, 16 female) in an elegantly designed study [63]. The participants were exposed one day to a stimulant odor (grapefruit, fennel, or black pepper EO), one day to no odor (control), and one day to a relaxant odor (lavender, patchouli, or rose EO) for 30 min in different combinations. Blood samples were taken before and after odor exposure, and various immunological markers (CXCL8, CCL3, CCL4, cortisol, PAI-1, TNFα, IL-6, eotaxin, CCL5, CXCL8 release, and CD16) and neutrophil activity were measured. No significant effect was observed with any EO for any parameter tested [63]. In a similar experimental design, 56 healthy volunteers were exposed to a relaxant odor (lavender, *Lavandula angustifolia* EO), to a stimulant odor (lemon, *Citrus limonum* EO), and to no-odor (water) for about 1.25 h during three separate visits in a blinded or primed (information was given on the applied EO) manner. IL-6 and IL-10 production of peripheral blood lymphocytes stimulated with concanavalin A did not alter significantly. DTH responses to *Candida*, which correspond to T-cell immunity, were actually larger in the no-odor group than in the groups receiving lemon or lavender. Lemon oil reliably enhanced positive mood independent of expectations or previous use of aromatherapy [65].

## 6. Effects of Essential Oils from Trees—Forest Bathing

Qing Li and colleagues examined the effects of different wood EOs or components on NK-92MI cells, an IL-2 independent human NK cell line [71]. After incubation of NK-92MI cells with different concentrations of *Chamaecyparis* stem oil, white cedar stem oil, 1,8-cineole, or α-pinene, for different durations, they significantly increased cytolytic activity of NK-92MI cells to K562 cells, in a dose- and time-dependent manner, and significantly increased the expression of perforin, granzyme A, and granulysin in the same cells. Furthermore, pretreatment of NK-92MI cells with *Chamaecyparis* stem oil and white cedar stem oil partially prevented the inhibition of NK activity induced by dimethyl 2,2-dichlorovinyl phosphate (DDVP), an organophosphorus pesticide, and also a range of wood EOs partially, but significantly, restored the DDVP-induced NK activity inhibition [71].

Such wood EOs are also present in the air of forests composed of the respective trees. We breathe in these EOs derived from trees while visiting a forest. To investigate if the human NK cell activity enhancing effect demonstrated in vitro could be observed in human subjects as well, Li and co-workers carried out a sequence of field experiments to study the effect of forest bathing [72]. A forest bathing trip is a short, leisurely visit to a forest for the purpose of relaxation and recreation, called “Shinrin-yoku” in Japanese. NK cell activity, the number of NK cells, the percentage, and number of perforin-, granulysin-, and granzyme A/B-expressing cells in the peripheral blood lymphocytes of the healthy male participants were found to become significantly increased even after a 2-h walk in a forest field on the first day of a three-day/two-night forest trip. After further two 2-h long walking sessions on the second day of the trip, the measured parameters further elevated compared to before the trip [73]. In a consecutive study, the increase of NK cell activity and of the numbers of cytolytic molecule expressing cells was found to last for more than seven days after the trip. In addition, a similar trip to a city without forests did not increase the measured immunological parameters [74]. Similar results were achieved with female subjects in a separate experiment [75]. NK activity and the expression of the cytolytic molecules were also enhanced after a one-day trip to a forest park that involved a morning and an afternoon 2-h long walking [76].

Han et al. also showed a significant increase in NK cell activity measured before and after a two-day forest therapy program in subjects with chronic widespread pain [77]. The difference was also significant when compared to a control group [77]. Another study aiming to assess the feasibility of adjuvant forest therapy also found enhanced natural cytotoxicity. Forest therapy, including a 2-h walk daily for 14 days, significantly increased the number of NK cells and the levels of perforin and granzyme B of 11 volunteer women with stage III breast cancer having completed standard treatments including surgery, radiotherapy, and chemotherapy [78].

In a work conducted by Im and colleagues among university students, serum IL-8 and TNFα levels were significantly decreased after a two-hour exposure to a forest environment compared to those after exposure to an urban environment suggesting a reduction in inflammation [79]. Mao and co-workers [80] demonstrated a significant reduction of the level of IL-6 in the sera of elderly patients with essential hypertension after a seven-day forest bathing trip with two, 1.5 h walks daily, compared to its baseline level. TNFα levels remained unaltered [80]. The levels of IL-6 were found to be significantly lower in the sera of elderly patients with chronic heart failure after participating in a four-day forest bathing trip, including two, 1.5 h walks daily, compared to the urban control group. There were no significant changes regarding TNFα [81].

Tsunetsugu et al. found no difference in IgA concentrations in the saliva of male participants measured before and after a 15-min walking at an unhurried pace in a forest site, or a 15-min watching of the scenery while sitting still on a chair [82].

Spending time in forest environments has other beneficial physiological and mental effects including, impact on cardiovascular, metabolic, endocrine, and mood parameters [83,84,85,86,87,88]. Breathing in the EOs from trees while staying in a forest is a possible mechanism that may induce the health benefits and immune function enhancement of forests and forest bathing, but other mediators may also give rise or contribute to such positive effects. The possible roles of visual, auditory and tactile experiences, biodiversity, negative air ions, reduced air pollution and heat, microbial input, stress reduction, relaxation, and some others are suggested and discussed in the literature [89,90,91,92]. Moreover, not only the presence of these factors but also the duration and frequency of exposure to outdoor green spaces influence the health-promoting effect [93,94].

## 7. Conclusions

There is a growing interest in the immunomodulatory effects of plant-derived compounds because of the increasing occurrence of diseases related to failing immunoregulation and the frequent toxicity and side effects of the currently used clinical immunomodulatory drugs. Furthermore, these compounds seem to have good efficacy in preventive medicine and in promoting a natural and healthy lifestyle. Research has focused on the anti-inflammatory effects of plants and Eos, where as the immunostimulatory effects have been investigated to a less extent. The immunomodulatory effects are mainly examined on a cellular (monocytes/macrophages, neutrophil granulocytes, NK cells, and T and B lymphocytes) and molecular level (cytokines, immunoglobulins), much less data is available in the literature on the potential molecular genetical mechanisms explaining such immunological effects. The possible regulating role of the transcription factor NF-κB has been presented [24]. Other plant-derived materials, such as the polyphenols, are able to modulate the activity of transcription factors NFκB, SREBP-1c, Nrf2, and PPAR-α/γ in mice/rats [95,96,97,98], which may guide investigations regarding volatile oils and their constituents as well.

Research on EOs supporting the immune system takes place predominantly in some countries of the Far and Middle East, in India, and in Brazil, where medicinal plants are traditionally used and valued. Only a couple of papers are accomplished in European countries, in the United States, Canada, or Australia. Researchers of the former countries characteristically prefer to study the EOs of the plants and herbs endemic to their region, resulting in fragmentary data on single EOs. Only few EOs are the subject of multiple studies offering sufficient data for evaluation. Eucalyptus and ginger EOs seem to have immune function enhancing or immune function balancing properties based on more studies, bearing in mind that the different types of immune cells or functions may be differentially influenced by the same EO or component. The majority of the works use whole EOs and not isolated constituents of EOs. While the exact compounds of EOs derived from the same plant species can be quite variable, synergistic activities may be present among the chemical constituents of the whole EOs, which are otherwise more available in practice and everyday life than purified components.

Future research should focus on the potential immune function enhancing effects of a less wide variety of EOs to provide a more convincing scientific evidence for each, including the potential molecular mechanisms lying in the background. Considering the results of special fields of EO application like the dietary supplementation for animals to improve health and forest bathing, an interdisciplinary approach with environmental and public health research, forestry, and animal husbandry is highly desirable.

## Figures and Tables

**Table 1:** Essential oils used in the experiments.

Essential Oils or EO Constituents Used in Different Experimental Settings	References
**In Vitro Investigations**	
*Allium sativum* (garlic)	[22]
*Eucalyptus globulus* (eucalyptus)	[23,24]
eugenol (from *Syzygium aromaticum*, clove)	[25,26]
*Ferula iliensis*	[27]
frankincense EO (derived from *Boswellia carterii*)	[28]
*Lavandula angustifolia* (lavender)	[29]
*Pituranthos tortuosus*	[30]
*Schinus molle*	[31]
*Syzygium cumini* (jambolão)	[32]
*Thymus vulgaris* (thyme)	[33]
**In Vivo Pre-Clinical Studies**	
*Carum copticum* (bishop’s weed) seeds EO compounds: α-pinene and carvacrol	[34]
*Eucalyptus globulus* (eucalyptus)	[23,24]
*Eugenia caryophyllata*	[35]
*Massoia aromatica* bark EO and massoialactone	[36,37]
*Melaleuca viridiflora* (niaouli)	[38]
orange EO, and its components: Limonene, linalool, and citral	[39]
*Panax ginseng* (ginseng)	[40]
*Salvia officinalis* (sage)	[41]
*Syzygium aromaticum* (clove)	[41]
*Zanthoxylum rhoifolium*	[42]
*Zingiber officinale* (ginger)	[41]
**Dietary Supplementation for Livestock and Fish**	
a blend of thyme (*Thymus vulgaris*), peppermint (*Mentha piperita*), and eucalyptus (*Eucalyptus globulus*)	[43]
a blend of thymol and carvacrol	[44]
a blend of thymol and cinnamaldehyde	[45,46,47,48]
*Allium sativum* (garlic)	[49]
*Ducrosia anethifolia*	[50]
*Eucalyptus globulus* (eucalyptus)	[51]
*Juniperus communis* (juniper)	[49]
*Ocimum americanum* (American basil)	[52]
*Origanum onites*	[53]
*Origanum vulgare* (oregano)	[54]
*Satureja hortensis* (savoury)	[55]
*Thymus vulgaris* (thyme)	[56]
**Human Studies**	
a blend of lavender (*Lavandula angustifolia*), cypress (*Cupressus sempervirens*), and sweet marjoram (*Origanum majorana*)	[57]
a blend of orange sweet (*Citrus sinensis*), true lavender, and marjoram sweet (*Origanum majorana*)	[58]
a blend of sweet orange (*Citrus aurantium*), lavender (*Lavandula angustifolia*), and sandalwood (*Santalum album*)	[59]
*Chamaecyparis obtusa* (hinoki cypress)	[60]
Citrus fragrance (lemon oil+orange oil+bergamot oil+*cis*-4-hexenol)	[61]
ginger EO	[62]
grapefruit, fennel, pepper, lavender, patchouli, and rose	[63]
*Lavandula angustifolia* (lavender)	[64]
*Lavandula angustifolia* (lavender), *Citrus limonum* (lemon)	[65]
